# Efficacy and safety of electroacupuncture treatment in the prevention of negative moods in healthy young men after 30 h of total sleep deprivation: study protocol for a single-center, single-blind, parallel-arm, randomized clinical trial

**DOI:** 10.1186/s13063-021-05659-x

**Published:** 2021-11-01

**Authors:** Bing Yan, Fu-chun Wang, Tian-shu Ma, Yan-ze Liu, Wu Liu, Lei Cheng, Zi-yuan Wang, Zhong-ke Wang, Cheng-yu Liu

**Affiliations:** 1 School of Acupuncture-Moxibustion and Tuina, Changchun University of Chinese Medicine, Changchun, China; 2 Department of Acupuncture, The Affiliated Hospital of Changchun University of Chinese Medicine, Changchun, China; 3 Innovative Practice Center, Changchun University of Chinese Medicine, Changchun, China; 4 School of Rehabilitation Medicine, Changchun University of Chinese Medicine, Changchun, China

**Keywords:** Sleep deprivation, Electroacupuncture, Mood, Prevention, Randomized controlled trials

## Abstract

**Background:**

Sleep deprivation (SD) among young adults is a major public health concern. In humans, it has adverse effects on mood and results in serious health problems. Faced with SD, persons may take precautionary measures to try and reduce their risk. The aim of this study is to evaluate the efficacy and safety of electroacupuncture (EA) for the prevention of negative moods after SD. In addition, we will do a comparison of the effects of EA on mood after SD at different time points.

**Methods:**

This randomized controlled trial (RCT) will be performed at the First Affiliated Hospital of Changchun University of Chinese Medicine in China. The Standards for Reporting Interventions in Clinical Trials of Acupuncture 2010 will be strictly adhered to. Forty-two healthy male volunteers will be distributed into acupoints electroacupuncture (AE) group, non-acupoints electroacupuncture (NAE) control group, or blank control group. This trial will comprise 1-week baseline (baseline sleep), 1-week preventative treatment, 30-h total sleep deprivation (TSD), and 24-h after waking follow-up period. Participants in the AE group and the NAE control group during the preventative treatment period will be administered with EA treatment once daily for 1 week. Participants in the blank control group will not be administered with any treatment. The primary outcome will be the Profile of Mood States (POMS) Scale. Secondary outcome measures will include changes in the Noldus FaceReader (a tool for automatic analysis of facial expressions) and Positive and Negative Affect Schedule (PANAS) Scale. Total sleep deprivation will be 30 h. During the 30-h TSD period, participants will be subjected to 11 sessions of assessment. Adverse events will be recorded.

**Discussion:**

This study is designed to evaluate the efficacy and safety of EA for the prevention of negative moods after SD. The results of this trial will allow us to compare the effects of EA on mood after SD at different time points. Moreover, the findings from this trial will be published in peer-reviewed journals.

**Trial registration:**

Chinese Clinical Trial Registry Chi2000039713. Registered on 06 November 2020

## Administrative information

Note: the numbers in curly brackets in this protocol refer to SPIRIT checklist item numbers. The order of the items has been modified to group similar items (see http://www.equator-network.org/reporting-guidelines/spirit-2013-statement-defining-standard-protocol-items-for-clinical-trials/).

**Table:** 

Title {1}	Efficacy and safety of electroacupuncture treatment in the prevention of negative moods among healthy young men after 30 h of total sleep deprivation: study protocol for a single-center, single-blind, parallel-arm, randomized clinical trial
Trial registration {2a and 2b}.	Chinese Clinical Trial Registry, Chi2000039713, registered on 06 November 2020.
Protocol version {3}	Protocol version V1.1 dated 3 December 2020
Funding {4}	This research is funded by Chengyu Liu, the National Natural Science Foundation of China (grant number: 81973954) and Scientific Research and Development Foundation of Changchun University of Chinese Medicine.
Author details {5a}	Bing Yan: School of Acupuncture-Moxibustion and Tuina, Changchun University of Chinese Medicine, Changchun, China Fu-chun Wang: Department of Acupuncture, The Affiliated Hospital of Changchun University of Chinese Medicine, Changchun, China Tian-shu Ma: Innovative Practice Center, Changchun University of Chinese Medicine, Changchun, China Yan-ze Liu: School of Acupuncture-Moxibustion and Tuina, Changchun University of Chinese Medicine, Changchun, China Wu Liu: School of Acupuncture-Moxibustion and Tuina, Changchun University of Chinese Medicine, Changchun, China Lei Cheng: School of Acupuncture-Moxibustion and Tuina, Changchun University of Chinese Medicine, Changchun, China Zi-yuan Wang: School of Acupuncture-Moxibustion and Tuina, Changchun University of Chinese Medicine, Changchun, China Zhong-ke Wang: School of Acupuncture-Moxibustion and Tuina, Changchun University of Chinese Medicine, Changchun, China Cheng-yu Liu: School of Rehabilitation Medicine, Changchun University of Chinese Medicine, Changchun, China
Name and contact information for the trial sponsor {5b}	Sponsor: The Affiliated Hospital of Changchun University of Chinese Medicine, Gongnong Road No.1478, Chaoyang District, Changchun, China Coordinating Investigator (contact): Prof. Fuchun Wang Acupuncture Department, The Affiliated Hospital of Changchun University of Chinese Medicine, Gongnong Road No.1478, Chaoyang District, Changchun, China Coordinating Investigator (contact email): fuchenwang420@126.com
Role of sponsor {5c}	The study sponsor and funder had no role in the design, collection, management, analysis, interpretation of data, decision to publish or the preparation of the manuscript, nor in the writing of the report.

## Introduction

### Background and rationale {6a}

Sleep is an essential part of life. In modern societies, SD is particularly prevalent because many sacrifice sleep to meet work demands and social commitments. Clinically, SD is defined as “abnormal sleep that can be described in measures of deficient sleep quantity, structure, and/or sleep quality” [1]. It is characterized by sleep loss, restricted sleep duration, or REM sleep exclusion and is a major public health issue [2]. In recent years, the prevalence of SD in the society has been on the rise [3], thereby creating a challenge in the management of daily performance deficits due to sleep loss. SD endangers individual health, both in acute and chronic states. It has been documented that SD influences emotions [4] and can result in impairments in affective states (e.g., increased anxiety, depressed mood, anger, tension, frustration, and irritability) [5, 6]. Acute experimental SD has been associated with decreased social emotional functions, such as slower voluntary facial responsiveness to emotional stimuli [7], less accuracy in emotional face recognition [8], and an increased negativity bias [9]. These studies imply that SD impairs emotional information processing or causes such processing to be negatively biased. Epidemiological studies have shown that poor sleep is prospectively correlated with psychiatric morbidity, especially depression [10], where emotional dysfunction forms part of the symptomatology. In summary, there is evidence that the perception of emotional faces, as well as facial expressions, might be altered after SD.

The damage to human health that is a result of early SD is difficult to detect, and the modern medical testing equipment makes it difficult to accurately diagnose it before it causes significant organic damage to the body. However, SD has a significant negative impact on mood. Given SD can cause serious harm to the human body, it is important to seek reliable countermeasures and preventative measures in cases where it cannot be avoided or where the disease has already occurred. Currently, there are no official guidelines for the treatment of SD. The usual treatment for SD is to examine one’s sleep hygiene in order to eliminate behavioral habits that adversely affect sleep. However, due to increased work pressure and the fast pace of life in modern society, this self-treatment method is difficult to implement. Therefore, chemotherapeutic options should be considered. Currently, modafinil is the only FDA-approved medication for the treatment of sleep disorders in shift work [11]. Appropriately administered medications such as caffeine, modafinil, and amphetamines, which excite the center, can have an anti-sleepy fatigue effect. Although these drugs have been shown to be effective in clinical use, they have also been associated with many adverse effects, such as sleep interference, mental disorders, and addiction. Moreover, when used as sleep aids, some of these drugs can exacerbate daytime fatigue if the dose is too high or if the half-life is too long. Sedative sleep aids can also worsen SD [12].

Researchers and patients are seeking alternative options, such as non-drug therapies, for controlling negative emotions caused by SD. Acupuncture, as a typical treatment modality of traditional Chinese medicine (TCM), has been applied for diseases for over 2000 years. Acupuncture is a well-known form of Asian medical treatment that is used not only as an effective curative method, but also to prevent illness and maintain health. Electroacupuncture stimulation, an application of electrical current on acupuncture needles, is one of the most popular types of this traditional therapy. Studies have aimed at elucidating the underlying mechanisms of acupuncture. Recent meta-analyses have documented that EA can alleviate symptoms of insomnia, regulate related negative emotions, and improve the quality of life in patients with insomnia [13, 14]. Moreover, several studies have now shown that acupuncture can have a good regulatory effect on mood [15–17]. However, the preventive effect of EA on negative emotions after SD should be further verified in clinical trials. Therefore, we aim at performing a single-center randomized controlled trial to investigate the efficacy and safety of EA in the prevention of negative emotions after SD.

### Objectives {7}

This study is designed to evaluate the efficacy and safety of EA for the prevention of negative moods after SD. Moreover, the effects of EA on mood after SD at different time points will be compared. The findings of this trial will provide more reliable evidence for clinical acupuncture prevention in the treatment of negative moods after SD.

### Trial design {8}

**Fig. 1:**
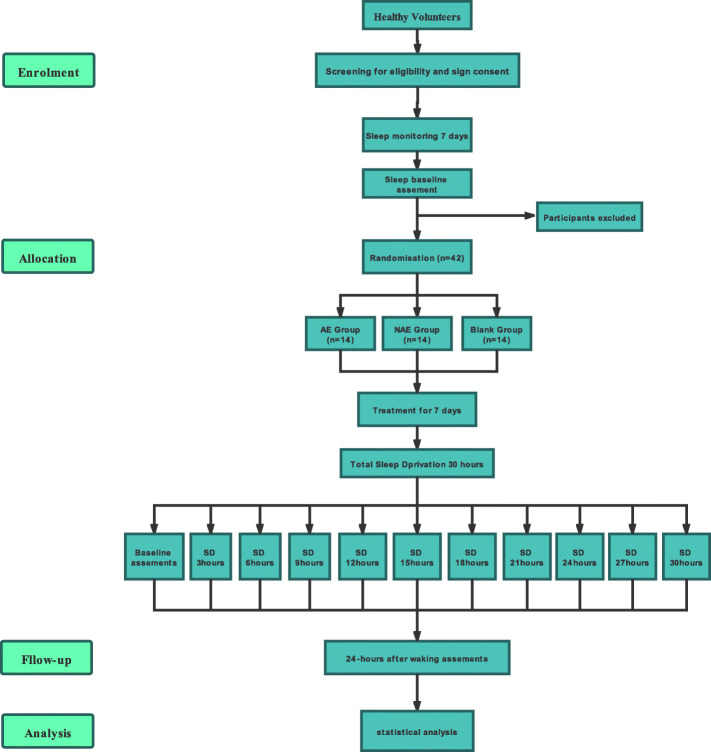
Flow diagram of the trial

This will be a single-center, single-blind, parallel-arm, randomized clinical trial. Forty-two healthy male volunteers will be allocated into the AE group, NAE group, or blank group with an allocation ratio of 1:1:1. This trial will comprise 1-week baseline (baseline sleep), 1-week preventative treatment, 30-h TSD, and 24-h after waking follow-up periods. Figure 1 shows the flow diagram of the trial.

## Methods: participants, interventions, and outcomes

### Study setting {9}

This RCT will be performed at the First Affiliated Hospital of Changchun University of Chinese Medicine, China. It is a comprehensive tertiary level Chinese medicine hospital with medical treatment, scientific research, prevention, and health care. The hospital has a clinical research center for acupuncture and moxibustion. This study will be conducted in accordance with the Declaration of Helsinki and has been approved by the Ethics Committee of the First Affiliated Hospital of Changchun University of Chinese Medicine (CCZYFYLL2020-054) and has been registered in the Chinese Clinical Trial Registry (ChiCTR2000039713).

### Eligibility criteria {10}

All participants will be subjected to a standardized interview and be given more information about this study. To minimize treatment bias, the acupuncturists who will be involved in this trial are specialists in acupuncture, with more than 5 years of experience in acupuncture treatment. They have valid acupuncture licenses (Chinese medicine practitioner license) from the Ministry of Health of the People’s Republic of China. Before performing this trial, the acupuncturists will receive special training regarding the purpose and content of the trial, treatment strategies, and quality control. In this study, we selected non-smoking men as the study subjects for this trial. Evidence indicates that women are more susceptible to the maladaptive emotional consequences of SD than men [18, 19]. The prevalence rates for both insomnia and anxiety disorders are higher in women relative to men [20, 21]. Women also demonstrate a stronger comorbidity of sleep disturbance and anxiety disorders than men [22]. However, there were also gender differences in the response to electroacupuncture treatment in subjects. In addition, nicotine consumption through tobacco products is highly comorbid with mood disorders, including depression, anxiety, and irritability. Tobacco use could precipitate mood dysregulation [23]. However, many studies have reported that nicotine can improve symptoms of depression under some conditions [24, 25]. This study is designed to evaluate the efficacy and safety of electroacupuncture treatment for the prevention of negative moods in healthy young men after SD. Therefore, we chose non-smoking males as the subjects for this trial.

### Inclusion criteria

The inclusion criteria will be as follows:

i Healthy male volunteers aged 18 to 30 years at the screening visit.
ii Participants must not have had any congenital defects or chronic diseases within the 3 years prior to the screening visit and should have no pathological or abnormal clinical findings on physical examination.
iii Participants must not have any sleep disorders and must have Pittsburgh Sleep Quality Index (PSQI) scores of 7 points or lower.
iv Participants must have Self-rating Anxiety Scale (SAS) scores of 50 points or lower and Self-rating Depression Scale (SDS) scores of 53 points or lower.
v Participants must keep normal nocturnal sleeping hours (between 21:00 and 09:00) for a week before the first clinical trials.
vi Participants must sleep for at least 7 h per night for a week before the first clinical trials.
vii Participants must be non-smokers or ex-smokers who stopped smoking at least 1 year before the screening visit.
viii An agreement to participate in the study by signing the informed consent.

### Exclusion criteria

Participants who experience, have, or have had one or more of the following will be excluded:

i History of regular alcohol consumption (> 210 g/week) within the 6 months before the screening visit (beer (5% v/v): 250 mL = 10 g, soju (20% v/v): 50 mL = 8 g, wine (12% v/v): 125 mL = 12 g)
ii Participation in another clinical study within 2 months before the screening visit
iii Intake of prescribed or over-the-counter drugs within 7 days before the first clinical trials
iv Participants who will have received acupuncture treatment during the 7 days before the first clinical trials
v Participants with a fear of acupuncture or infection related to the locations of Baihui (GV-20), Shenmen (HT-7), and Sanyinjiao (SP-6)

### Who will take informed consent? {26a}

Personal information will be collected after oral and written consents are obtained from each participant at the first visit. After potential participants voluntarily consent to the study, they will be screened using pre-determined inclusion/exclusion criteria at the first visit. Written informed consent will be obtained from all the participants by the principal investigator or sub-investigators prior to enrollment.

### Additional consent provisions for collection and use of participant data and biological specimens {26b}

This will not be applicable as no biological specimens will be collected for research purposes.

### Interventions

The two control interventions will be NAE and the blank control. The manipulation of NAE will be conducted as described below. To eliminate out placebo effects, the NAE control group will be used. The blank control group will be used to assess whether EA is effective. Intergroup comparisons will be done for the three groups.

#### Intervention description {11a}

Interventions will be according to the records in ancient books and research results of modern papers about treating diseases related to sleep disorders with acupuncture in China or in the West [26–29]. These interventions will be slightly modified by Chinese acupuncturists and acupuncture experts from the study team and non-study team in all regions where the study will be conducted. The project team has made SOPs for clinical research to ensure that the treatment is followed throughout the clinical research. Participants will be randomly allocated into one of three groups: the AE group, the NAE control group, or the blank control group.

#### The acupoints electroacupuncture group

**Fig. 2:**
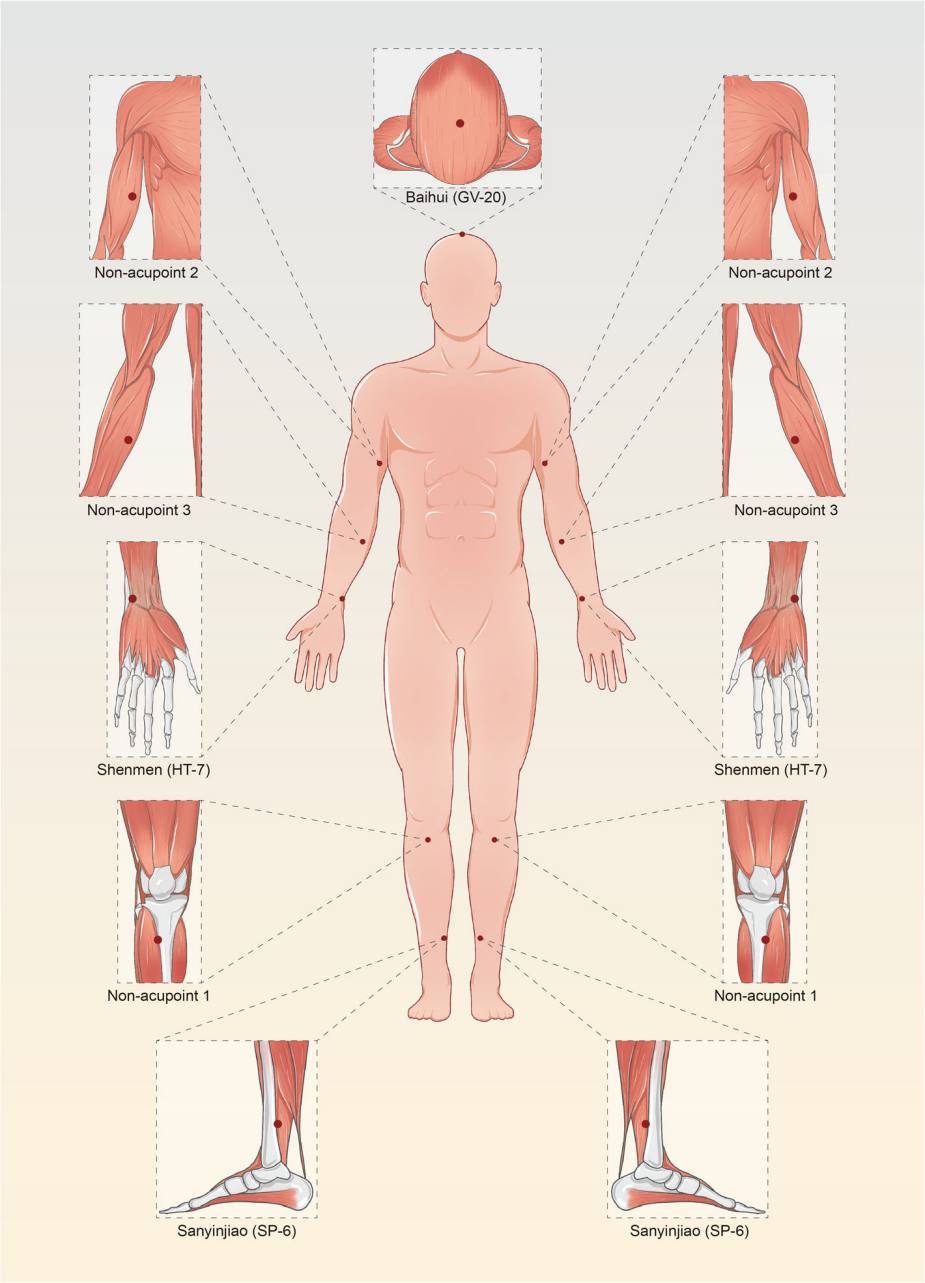
All acupoints

The acupoints to be used in the AE group are Baihui (GV-20), Shenmen (HT-7), and Sanyinjiao (SP-6), which are recommended points for the treatment of diseases related to sleep disorders [30, 31]. HT-7 is the point with the highest frequency of use [32], and SP-6 and GV-20 are often used with HT-7 as a combination regimen according to the TCM theory and clinical experience of acupuncture therapists [33]. The acupuncture operation is based on the textbook of the 13th Five-Year Plan of the Ministry of Health. Participants will be required to take the supine position. Acupoints will be needled after disinfection. Baihui (GV-20) will be horizontally punctured 0.3–0.5 cun (the acupuncture needle tip travels in the opposite direction to the meridian circulation of Dumai meridians). Shenmen (HT-7) will be perpendicularly punctured 0.5–0.8 cun while Sanyinjiao (SP-6) will be perpendicularly punctured 1.0–1.5 cun. All acupoints will be localized according to the WHO Standard Acupuncture Locations as shown in Table 1 and Fig. 2.

**Table 1:** Locations of acupoints for the acupoint EA and non-acupoint EA groups

Group	Acupoint	Location	Descriptions
1	Baihui (GV-20)	At the vertex on the midline, in the depression 5 cun posterior to the anterior hairline and 7 cun superior to the posterior hairline	Punctured horizontally 0.3–0.5 cun
	Shenmen (HT-7)	At the wrist joint, on the radial side of flexor carpi ulnaris, in the depression at the proximal border of the pisiform bone	Punctured perpendicularly 0.5–0.8 cun
	Sanyinjiao (SP-6)	On the medial side of the lower leg, 3 cun superior to the prominence of the medial malleolus, in a depression close to the medial crest of the tibia	Punctured perpendicularly 1.0–1.5 cun
2	Non-acupoint 1	On the edge of the tibia 1 to 2 cm lateral to the Zusanli (ST36) horizontally	Punctured perpendicularly 0.5–1 cun
	Non-acupoint 2	On the half between the tip of the elbow and the axilla	Punctured perpendicularly 0.5–1 cun
	Non-acupoint 3	On the ulnar side, half between the epicondylus medialis of the humerus and the ulnar side of the wrist	Punctured perpendicularly 0.5–1 cun

Group 1: acupoints electroacupuncture group

Group 2: non-acupoints electroacupuncture control group

^a^1 cun (≈ 20 mm) is defined as the width of the interphalangeal joint of the patient’s thumb

#### Non-acupoint electroacupuncture control group

In the NAE control group, we will select non-acupoints as reported in previous studies, including non-acupoint 1, non-acupoint 2, and non-acupoint 3. Non-acupoint 1, the edge of the tibia 1 to 2 cm lateral to the Zusanli (ST-36) horizontally [34], will be perpendicularly punctured 0.5–1 cun. Non-acupoint 2, half between the tip of the elbow and the axilla [35], will be perpendicularly punctured 0.5–1 cun. Non-acupoint 3, the ulnar side, half between the epicondylus medialis of the humerus and the ulnar side of the wrist [35], will be perpendicularly punctured 0.5–1 cun. All non-acupoints are shown in Table 1 and Fig. 2.

In each session, all acupoints and non-acupoints will be bilaterally punctured by filiform needles. After the puncture of 3 acupoints or non-acupoints by needles, 6 auxiliary needles will be punctured at 2 mm lateral to every acupoint or non-acupoint and punctured to a depth of 2 mm without manual stimulation. Transcutaneous electric acupoint stimulation (Hwato SDZ-V Acupoint Nerve Stimulator, Suzhou Medical Co., Ltd.) will be used for electro-acupuncture stimulation at every acupoint or non-acupoint after needle insertion. Each acupuncture needle at the acupoints or non-acupoints and auxiliary needles will be connected to the electricity by Hwato SDZ-V for 30 min. The stimulation frequency will be 2/100 Hz. The stimulation intensity will vary from 0.1 to 1.0 mA until the patients feel comfortable. The Massachusetts General Hospital Acupuncture Sensation Scales (MASS) will be used to determine whether the De-Qi sensations are produced during acupuncture. To screen for patients with the De-Qi sensation. The intensity of soreness, numbness, heaviness, warmth, cold, sharp pain, and dull pain will be rated on a numerical scale of 1–10.

The filiform needles to be used in this trial are single-use sterile acupuncture needles, namely Hwato Needles, made in Suzhou, China, 25–40 mm in length and 0.25 mm in diameter. Needles with a length of 13 mm and a diameter of 0.18 mm will be used as auxiliary needles without manipulation. De-Qi sensation will be achieved in the acupoints of the AE group by lifting and thrusting combined with twirling and rotating the needles. However, De-Qi sensation will not be achieved in the non-acupoints of the control group by the same needle manipulation methods. Needles will be retained in the AE group and the NAE control group for 30 min; afterwhich, the acupoint holes will be closed using clean cotton balls to avoid bleeding when withdrawing the needle. Participants in the two groups will be administered with 7 treatments over a period of 1 week, once a day.

#### Blank control group

Participants in the blank control group will not be given any treatment.

### Sleep deprivation procedures

The SD trial will be performed at the Clinical Centre for Acupuncture and Moxibustion at the First Affiliated Hospital of Changchun University of Chinese Medicine, China. Sleep deprivation will start at 7:00 am (baseline). Total sleep deprivation will be 30 h. During their free time, participants will spend most of their time in a large common room with both natural and electric lighting. During SD, participants will be permitted to play games, read, review school work, and use their personal electronic devices for entertainment. They will also be allowed to freely interact with each other and with the research staff. Napping, caffeinated beverages, and strenuous physical activity will be prohibited. Participants will be served breakfast, lunch, and dinner in the hospital cafeteria, with free access to snacks during their free time in the common room. Throughout the trial, participants will be constantly supervised by the researcher to ensure they do not fall asleep.

### Criteria for discontinuing or modifying allocated interventions {11b}

Participants will be allowed to leave the study at any time for any reason if they wish to, without any consequences. Participation in this study will also be ended by the investigator if the participant is uncooperative and/or does not attend study visits. This study will be prematurely ended in case of any adverse events or procedural-related complications or if the independent specialist in acupuncture and moxibustion advises its termination. The criteria for study termination will also include any serious adverse event (SAE).

### Strategies to improve adherence to interventions {11c}

All acupuncture treatments and laboratory tests will be provided for free to improve adherence to the intervention protocol. All subjects will be required to sign a written informed consent before participating and will be compensated monetarily. Participant and acupuncturist signatures will be required after each acupuncture session to monitor adherence.

### Relevant concomitant care permitted or prohibited during the trial {11d}

To ensure that participants within the three groups get adequate sleep (at least 7 h per night for a week before the first clinical trial) during the sleep baseline period, we will use sleep logs and actigraph (an automatic-scoring actigraph for measuring sleep in healthy adults) for recording. Throughout the study, participants will wear an actigraph (Micro Motionlogger SleepWatch®, Ambulatory Monitoring, Inc.; AMI) and report their bedtime, sleep onset latency, and wake time on a daily sleep diary. Alcoholic drinks, caffeinated beverages, prescription, over-the-counter medicines, and other preventive interventions will be prohibited during the trial.

### Provisions for post-trial care {30}

If the condition of a subject worsens after the trial or is accompanied by severe complications or serious adverse reactions, the specialist will take medical measures to deal with the harm based on the subject’s condition. The treatments will be free.

### Outcomes {12}

#### Primary outcome

##### Profile of Mood States Scale

The POMS [36] is a well-validated, 65-item self-report measure comprising six subscales: tension/anxiety, depression/dejection, anger/hostility, energy/activity, fatigue/vigor, and confusion/bewilderment. Participants will report on their mood using a 5-point scale (“not at all” to “extremely”).

#### Secondary outcomes

##### Positive and Negative Affect Schedule Scale

The PANAS [37] will be used to assess the positive and negative effects. Participants will be shown 20 adjectives with 10 describing positive moods and 10 describing negative moods. They will respond using a 5-point Likert scale (1 very slightly, 5 extremely).

##### Noldus FaceReader

The Noldus FaceReader software (FR; version 7.0, Noldus Information Technology) [38] is a facial analysis program that detects emotional facial expressions in film and photographs. The FaceReader can detect six basic emotions, happiness, sadness, anger, fear, surprise, and disgust, as well as neutral states. The FaceReader can also analyze the valence of facial expressions as well as the general state of arousal. The video stimuli are analyzed frame-by-frame to detect the intensity by which each of the six basic emotions is expressed on a scale of 0 to 1, where 0 indicates the absence of emotion while 1 indicates maximum intensity. In this study, the focus will be on the participants’ emotions after sleep deprivation. Prior to analysis, all videos will be calibrated to each participant’s own neutral expression to achieve the most accurate results. The videos will then be analyzed using the batch analysis mode, which sequentially analyzes each video without the need for supervision. Videos of participants’ facial expressions will be recorded using Logitech HDC 920 PRO Webcam, which will be placed above the computer screen. The videos will be processed using the Noldus FaceReader software and Observer XT (version 12.5, Noldus Information Technology). The software’s artificial intelligence is trained to register the activation of 20 action units (AUs) and to indicate scores for happiness, surprise, anger, sadness, disgust, and scared faces as proposed by the basic emotion framework.

### Participant timeline {13}

**Fig. 3:**
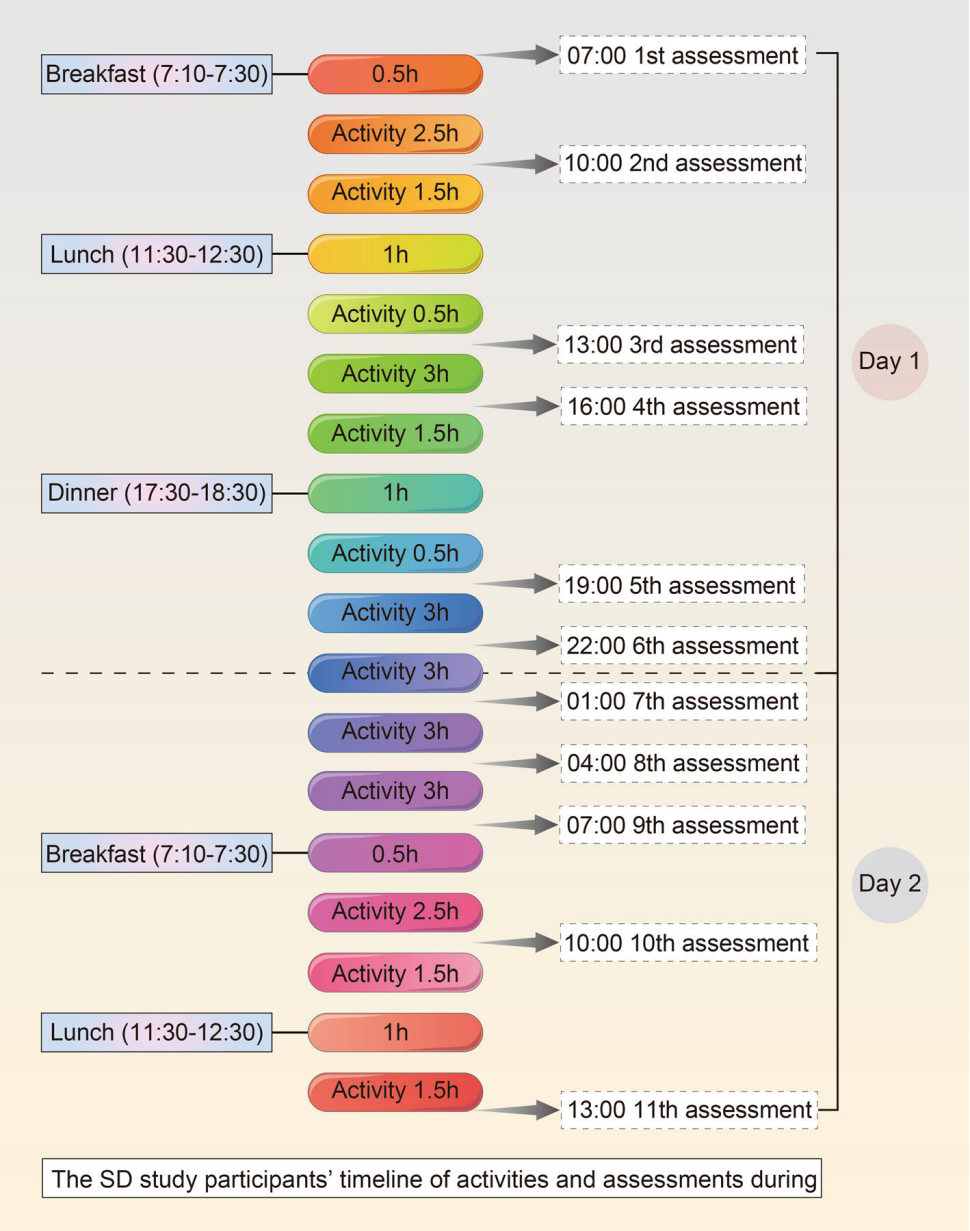
The SD study participants’ timeline of activities and assessments during hospitalization

The trial will comprise 1-week baseline, 1-week preventative treatment, 30-h TSD, and 24-h after waking follow-up periods. During the 30-h TSD period, participants will be assessed every 3 h for a total of 11 sessions. They will be evaluated at 7:00 am (baseline), 10:00 am, 1:00 pm, 4:00 pm, 7:00 pm, 10:00 pm, and on the next day at 1:00 am, 4:00 am, 7:00 am, 10:00 am, and 13:00 pm. AE will also be recorded. Table 2 shows the participant timeline. The SD study participants’ timeline of activities and assessments during hospitalization is shown in Fig. 3.

**Table 2:**
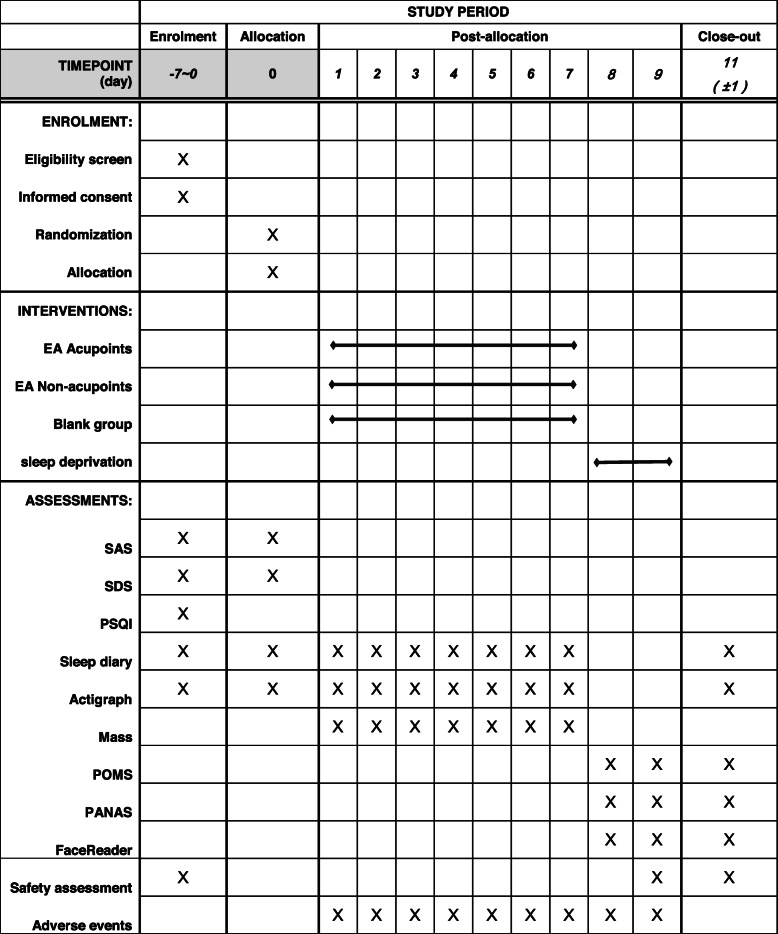
The schedule of enrollment, interventions, and assessments

The safety assessment comprises a routine blood test, routine urine test, routine feces test, kidney function test, liver function test, and electrocardiogram

*SAS* Self-Rating Anxiety Scale, *SDS* Self-Rating Depression Scale, *PSQI* Pittsburgh Sleep Quality Index, *POMS* Profile of Mood States Scale, *PANAS* Positive and Negative Affect Schedule Scale, *EA* electroacupuncture treatment, *Mass* Massachusetts General Hospital Acupuncture Sensation Scales

### Sample size {14}

This is an RCT study with three groups: an AE group, a NAE group, and a blank control group. The POMS will be the primary outcome measure observed. According to the findings of the clinical pre-trial study, the means of the POMS scores for each group are 8.26, 18.25, and 15.24, with standard deviations of 3.18, 5.96, and 5.42, respectively. The sample size was calculated by the PASS software (version 15.0.5, NCSS, LLC) [39], and at least 33 subjects will be needed in our study if the power is 0.9 and *α* is set to 0.05. To compensate for a 20% loss to follow-up, the sample size will be increased to 42 participants, of which at least 14 participants will be required in each group.

### Recruitment {15}

Several strategies will be applied in participant recruitment. Forty-two healthy male volunteers will be recruited by advertisement on notice boards at local Chinese universities. Advertisements will be placed across WeChat and websites. The posters and advertisements will contain brief introductions about the population to be enrolled, the free acupuncture treatments offered to volunteers, and the contact information of the researchers. All subjects will be required to sign a written informed consent before participating and will be monetarily compensated.

### Assignment of interventions: allocation

#### Sequence generation {16a}

Randomization will be done using the Excel random number method. The participating volunteers will be strictly screened according to the inclusion and exclusion criteria. Once inclusion is confirmed, participants will be numbered in the order by which they will be included. Random numbers will be generated by the rand function using Excel numbering after which they will be fixed and reordered.

#### Concealment mechanism {16b}

Randomization will be performed by a person who will not be engaged in the study in order to blind the identity of the participants.

#### Implementation {16c}

The randomization sequence will be generated using a random number table by an independent statistician. A sealed envelope will be used to hide the group assignments. Then, the envelopes will be numbered in sequential order and stored by a research assistant who will not be involved in the enrollment process. When an eligible volunteer is enrolled in the study, an envelope will be opened by research assistants that will be responsible for enrolling the volunteers.

### Assignment of interventions: blinding

#### Who will be blinded {17a}

Blinding methods will be applied to data statisticians and outcome evaluators throughout the trial. They will not be able to obtain information regarding the grouping and treatment of participants. Acupuncturists will not be masked due to the nature of acupuncture. However, acupuncturists will not participate in the data collection, statistics, and final outcome evaluation. For the intervention, different volunteers will be assigned to different treatment rooms with independent beds so as to avoid contact among subjects. Personnel in charge of random grouping, acupuncture treatment, data processing, and outcome evaluation will be completely separated so as to ensure the authenticity of the outcome of the trial to the greatest possible extent.

#### Procedure for unblinding if needed {17b}

At the end of the trial, we will first perform a first blinded unblinding to clarify the differences between the 3 groups, but will not know the content of the interventions in the 3 groups, on the basis of which statistical analysis will be performed. A second unblinding will then be performed, which will reveal which group is the AE group, which group is the NAE control group, and which group is the blank control group, to further analyze the results of the clinical trial. However, researchers will be permitted to be unblinded before the end of this trial in some specific situations, such as participants experiencing serious adverse events or disease progression, and where prompt treatment and appropriate management are needed. The procedure for unblinding will be as follows: (1) prior to unblinding, the researcher will need to inform the trial steering committee; (2) the researcher should unblind the intervention information of the patient based on the emergency envelope; and (3) the researcher will need to fill out the report form of unblinding and note it on the case report form (CRF).

### Data collection and management

#### Plans for assessment and collection of outcomes {18a}

Prior to the start of the trial, all data assessors will be uniformly trained in order to improve the quality of the data assessment. The primary outcome measure will be obtained using POMS while the secondary outcome measures will be obtained using PANAS and FaceReader. These three instruments have been widely used in trials related to the detection of mood changes [36–38]. During the 30-h TSD period, participants will be assessed every 3 h for a total of 11 assessments. During the 24-h after waking follow-up period, participants will also be assessed once.

#### Plans to promote participant retention and complete follow-up {18b}

During the recruitment period, participants will receive extensive information about the clinical trial requirements. The importance of completion of the follow-up will be stressed. Participants will be allowed to discontinue the study at any time and will not be obliged to give a reason for their discontinuation. Throughout the study, participants will be reminded to fill out the questionnaires during study visits, while during the follow-up period, investigators will check the responses and if necessary contact the participants for completion of their follow-up.

#### Data management {19}

A special data collector will record the participants’ personal data in the CRF. At least 5 completed CRFs from each clinical trial center shall be sent to the data manager in a timely fashion by the supervisor so as to establish a corresponding database. Epidata 3.1 (EpiData Association, Denmark), which is a statistical package, will be used for data entry. All data will be double entered using the data entry program run on a software platform. The data administrator shall ensure that the data in the CRF forms are completely and truly entered into the computer.

#### Confidentiality {27}

Only researchers will have access to the personal data in the trial. These data will not be published, and they will be discarded after the publication of the results.

#### Plans for collection, laboratory evaluation, and storage of biological specimens for genetic or molecular analysis in this trial/future use {33}

This is not applicable. Researchers confirm that there will be no laboratory and storage of biological specimens for genetic or molecular analysis in this study.

## Statistical methods

### Statistical methods for primary and secondary outcomes {20a}

Descriptive statistics will be used to describe the study population. Comparisons of the study groups by age, gender, and outcome characteristics will be done using analysis of variance (ANOVA) or chi-square tests. Depending on whether the data are normally distributed, the continuous variables will be described as the mean (standard deviation) or the median. One-way analysis of variance (ANOVA) will be used for comparisons among the three groups. Continuous variables, including the POMS scores, PANAS scores, and FaceReader, will be compared among the three groups at all time points using ANOVA. The Student-Newman-Keuls method will be used for pairwise comparisons. Adverse events will be summarized for each group and compared using Fisher’s exact tests. Statistical analyses will be performed using SAS 9.1.3. Statistical significance will be set at *p* ≤ 0.05.

### Interim analyses {21b}

There are no interim analyses planned.

### Methods for additional analyses (e.g., subgroup analyses) {20b}

There are no interim analyses planned.

### Methods in analysis to handle protocol non-adherence and any statistical methods to handle missing data {20c}

The intention-to-treat analysis will be used for all allocated participants in the baseline condition. Baseline characteristics will be summarized across the treatment groups. Missing values will be imputed by the last observation carried forward (LOCF) method.

### Plans to give access to the full protocol, participant level-data, and statistical code {31c}

Information from the full protocol will be published in a peer-reviewed journal. The relevant data obtained from this study protocol will be available upon reasonable request from the corresponding author.

### Oversight and monitoring

#### Composition of the coordinating center and trial steering committee {5d}

This is a monocenter study designed, performed, and coordinated at the First Affiliated Hospital of Changchun University of Chinese Medicine. Day-to-day support for the trial will be provided by the following:

Principle investigator: supervise the trial and take medical responsibility of the participants

Data manager: organize data collection and safeguard its quality

Study coordinator: trial registration, coordinate study visits, and document annual safety reports

Study physician: identify potential recruits, administer informed consent, and ensure follow-up according to the protocol

The study team will have biweekly meetings. There will be no trial steering committee or stakeholder and public involvement group.

#### Composition of the data monitoring committee, its role, and reporting structure {21a}

In accordance with the requirements of the standard for quality control and quality assurance of clinical research in TCM, a 4-level quality supervision system will be established. One supervisor will be appointed and a supervision committee set up to regularly monitor the data collected in clinical research, so as to ensure the authenticity and reliability of the project. The supervision team will supervise the entire process of information collection and input.

#### Adverse event reporting and harms {22}

Any adverse events reported by the participants will be recorded in the CRF, including the time, symptoms, signs, degree, duration, laboratory test index, treatment and outcome, follow-up, and follow-up time. Common treatment-related adverse events to be tested will include subcutaneous hematoma, continuous post-needling pain, itching at the sites of the needle insertion, and dizziness.

#### Frequency and plans for auditing trial conduct {23}

The Ethics Committee of the First Affiliated Hospital of Changchun University of Chinese Medicine will be responsible for the monitoring. Every 2 days, a report will be sent to the auditor. The process will be independent from investigators and the sponsor.

#### Plans for communicating important protocol amendments to relevant parties (e.g., trial participants, ethical committees) {25}

All research group members may introduce protocol amendments. These are then considered together, and the principal investigator will be responsible for the final decision to amend and how the substantive changes are communicated to the relevant stakeholders (the Ethics Committee of the First Affiliated Hospital of Changchun University of Chinese Medicine and ClinicalTrials.gov register). The protocol version with a date and list of amendments is clearly presented in the protocol.

#### Dissemination plans {31a}

We will share the results of this study with the key stakeholders through presentations in related seminars and publications in peer-reviewed journals.

## Discussion

Sleep time and emotional health are correlated [4]. In experimental studies on SD, the main results have centered on an increase in the negative effects of SD, with an increasing number of experimental studies preferring to focus on the negative emotions associated with SD [40, 41]. It has also been found that the negative effects of SD on mood are stronger than those on cognition or movement [42]. As a key component of traditional Chinese medicine, acupuncture is a clinically proven medical treatment option for many diseases. Moreover, it has preventative effects as it balances the body, allowing it to self-regulate. To the best of our knowledge, there are no similar RCTs regarding EA in the prevention of negative moods after SD. This will be a strictly designed trial aimed at evaluating the effects of EA in the prevention of negative moods after SD. Moreover, this study is aimed at overcoming some existing limitations, including illogical design, imperfect blinding method, and practical difficulties in previous acupuncture clinical studies. In the pre-trial phase, we were able to determine the scientific validity of the acupuncture treatment point selection protocol after discussions with several acupuncture clinical experts and through preliminary clinical pre-tests. In order to avoid variations in the acupuncture procedure, EA will be used to achieve good control of the depth, frequency, and duration of treatment.

This study has some limitations. First, the design is a single-blind trial. When participants sign the informed consent form, they are informed that this study is aimed at elucidating the efficacy of three interventions, and participants will be randomly assigned to any one of the groups. Due to the nature of acupuncture, masking acupuncturists is quite difficult. Therefore, 3 groups of participants will be allocated in different treatment rooms, and each participant will be separately treated to avoid communication between the participants in the group. Second, this is a single-center trial in China, and whether the results will have generalized significance for other subjects is unknown. The study has some limitations in terms of the selection of subjects. Studies [43, 44] have shown that older people are less emotionally affected by SD than younger people and that the negative effects of SD are stronger in younger people than in older people. Moreover, the effects are greater among women than in men. Therefore, in order to exclude age and gender effects, only healthy adult males (18–30 years) will be selected for our study. At the end of this trial, we hope that the results will provide more reliable evidence for clinical acupuncture prevention in the treatment of negative moods after SD.

## Trial status

Protocol version V1.1 dated 3 December 2020. The first participant was enrolled on 20 November 2020 and is still ongoing. Recruitment will be complete on 20 May 2021.

## Abbreviations

SD: Sleep deprivation
CRF: Case report form
LOCF: Last observation carried forward
ANOVA: Analysis of variance
AU: Action unit
SAE: Serious adverse event
MASS: Massachusetts General Hospital Acupuncture Sensation Scales
SDS: Self-Rating Depression Scale
SAS: Self-rating Anxiety Scale
PSQI: Pittsburgh Sleep Quality Index
EA: Electroacupuncture
TCM: Traditional Chinese medicine
TSD: Total sleep deprivation
RCT: Randomized controlled trial
AE: Acupoints electroacupuncture
NAE: Non-acupoints electroacupuncture
POMS: Profile of Mood States
PANAS: Positive and Negative Affect Schedule

## References

[1] Krause AJ, Simon EB, Mander BA, Greer SM, Saletin JM, Goldstein-Piekarski AN, Walker MP (2017). The sleep-deprived human brain. Nat Rev Neurosci.

[2] Colten HR, Altevogt BM, Institute of Medicine Committee on Sleep M, Research (2006). The National Academies Collection: reports funded by National Institutes of Health. Sleep disorders and sleep deprivation: an unmet public health problem.

[3] Hinz A, Glaesmer H, Brähler E, Löffler M, Engel C, Enzenbach C, Hegerl U, Sander C (2017). Sleep quality in the general population: psychometric properties of the Pittsburgh Sleep Quality Index, derived from a German community sample of 9284 people. Sleep Med.

[4] Baglioni C, Spiegelhalder K, Lombardo C, Riemann D (2010). Sleep and emotions: a focus on insomnia. Sleep Med Rev.

[5] JrLeDuc PA, Caldwell JA, Ruyak PS (2000). The effects of exercise as a countermeasure for fatigue in sleep-deprived aviators. Mil Psychol.

[6] Mougin F, Bourdin H, Simon-Rigaud ML, Didier JM, Toubin G, Kantelip JP (1996). Effects of a selective sleep deprivation on subsequent anaerobic performance. Int J Sports Med.

[7] Schwarz JFA, Popp R, Haas J, Zulley J, Geisler P, Alpers GW, Osterheider M, Eisenbarth H (2013). Shortened night sleep impairs facial responsiveness to emotional stimuli. Biol Psychol.

[8] Cote KA, Mondloch CJ, Sergeeva V, Taylor M, Semplonius T (2013). Impact of total sleep deprivation on behavioural neural processing of emotionally expressive faces. Exp Brain Res.

[9] Daniela T, Alessandro C, Giuseppe C, Fabio M, Cristina M, Luigi DG, Michele F (2010). Lack of sleep affects the evaluation of emotional stimuli. Brain Res Bull.

[10] Baglioni C, Riemann D (2012). Is chronic insomnia a precursor to major depression? Epidemiological and biological findings. Curr Psychiatry Rep.

[11] Sheng P, Hou L, Wang X, Wang X, Huang C, Yu M, Han X, Dong Y (2013). Efficacy of modafinil on fatigue and excessive daytime sleepiness associated with neurological disorders: a systematic review and meta-analysis. PLoS One.

[12] Abrams RM (2015). Sleep deprivation. Obstet Gynecol Clin North Am.

[13] Zhang M, Zhao J, Li X, Chen X, Xie J, Meng L, Gao X (2019). Effectiveness and safety of acupuncture for insomnia: protocol for a systematic review. Medicine..

[14] Chen B, Zhang G, Liu C, Chen Q, Zhang M, Li J, Zhou P, Fu W, Zhu M (2018). Effectiveness and safety of warm needle acupuncture on insomnia: protocol for a systematic review and meta-analysis. Medicine..

[15] Bosch P, van den Noort M, Yeo S, Lim S, Coenen A, van Luijtelaar G (2015). The effect of acupuncture on mood and working memory in patients with depression and schizophrenia. J Integr Med.

[16] Bussell J (2013). The effect of acupuncture on working memory and anxiety. J Acupunct Meridian Stud.

[17] Aoyama N, Fujii O, Yamamoto T (2017). Efficacy of parietal acupoint therapy: scalp acupuncture for neck/shoulder stiffness with related mood disturbance. Med Acupunct.

[18] van der Helm E, Gujar N, Walker MP (2010). Sleep deprivation impairs the accurate recognition of human emotions. Sleep..

[19] Sagaspe P, Sanchez-Ortuno M, Charles A, Taillard J, Valtat C, Bioulac B, Philip P (2006). Effects of sleep deprivation on Color-Word, Emotional, and Specific Stroop interference and on self-reported anxiety. Brain Cogn.

[20] Zhang B, Wing YK (2006). Sex differences in insomnia: a meta-analysis. Sleep..

[21] Kessler RC, McGonagle KA, Zhao S, Nelson CB, Hughes M, Eshleman S, Wittchen HU, Kendler KS (1994). Lifetime and 12-month prevalence of DSM-III-R psychiatric disorders in the United States. Results from the National Comorbidity Survey. Arch Gen Psychiatry.

[22] Johnson EO, Roth T, Breslau N (2006). The association of insomnia with anxiety disorders and depression: exploration of the direction of risk. J Psychiatr Res.

[23] Boden JM, Fergusson DM, Horwood LJ (2010). Cigarette smoking and depression: tests of causal linkages using a longitudinal birth cohort. Br J Psychiatry.

[24] Salín-Pascual RJ, de la Fuente JR, Galicia-Polo L, Drucker-Colín R (1995). Effects of transderman nicotine on mood and sleep in nonsmoking major depressed patients. Psychopharmacology (Berl).

[25] Tizabi Y, Overstreet DH, Rezvani AH, Louis VA, Clark E, Janowsky DS (1999). Antidepressant effects of nicotine in an animal model of depression. Psychopharmacology (Berl).

[26] Zhang J, He Y, Huang X, Liu Y, Yu H (2020). The effects of acupuncture versus sham/placebo acupuncture for insomnia: a systematic review and meta-analysis of randomized controlled trials. Complement Ther Clin Pract.

[27] Bosch P, van den Noort M, Staudte H, Lim S (2015). Schizophrenia and depression: a systematic review of the effectiveness and the working mechanisms behind acupuncture. Explore (NY).

[28] Huo ZJ, Guo J, Li D (2013). Effects of acupuncture with meridian acupoints and three Anmian acupoints on insomnia and related depression and anxiety state. Chin J Integr Med.

[29] Spence DW, Kayumov L, Chen A, Lowe A, Jain U, Katzman MA, Shen J, Perelman B, Shapiro CM (2004). Acupuncture increases nocturnal melatonin secretion and reduces insomnia and anxiety: a preliminary report. J Neuropsychiatry Clin Neurosci.

[30] Yin X, Gou M, Xu J, Dong B, Yin P, Masquelin F, Wu J, Lao L, Xu S (2017). Efficacy and safety of acupuncture treatment on primary insomnia: a randomized controlled trial. Sleep Med.

[31] Shergis JL, Ni X, Jackson ML, Zhang AL, Guo X, Li Y, Lu C, Xue CC (2016). A systematic review of acupuncture for sleep quality in people with insomnia. Complement Ther Med.

[32] Huang K, Liang S, Xu Y, Lu S (2015). Law of acupoint selection in acupuncture treatment for insomnia based on data mining method. Zhongguo Zhen Jiu.

[33] Wang J, Wang J, Wang L, Zhang Y (2015). Senile insomnia treated with integrated acupuncture and medication therapy: a randomized controlled trial. Zhongguo Zhen Jiu.

[34] Chang CH, Huang JL, Ting CT, Chang CS, Chen GH (2005). Atropine-induced HRV alteration is not amended by electroacupuncture on Zusanli. Am J Chin Med.

[35] Brinkhaus B, Hummelsberger J, Kohnen R, Seufert J, Hempen CH, Leonhardy H, Nogel R, Joos S, Hahn E, Schuppan D (2004). Acupuncture and Chinese herbal medicine in the treatment of patients with seasonal allergic rhinitis: a randomized-controlled clinical trial. Allergy..

[36] McNair DM, Lorr M, Droppleman IF (1971). Manual for the profile of mood states.

[37] Watson D, Clark LA, Tellegen A (1988). Development and validation of brief measures of positive and negative affect: the PANAS scales. J Pers Soc Psychol.

[38] Höfling TTA, Gerdes ABM, Föhl U, Alpers GW (2020). Read my face: automatic facial coding versus psychophysiological indicators of emotional valence and arousal. Front Psychol.

[39] NCSS Statistical Software. https://www.ncss.com/software/pass/. Accessed 7 Dec 2020.

[40] Kahn M, Sheppes G, Sadeh A (2013). Sleep and emotions: bidirectional links and underlying mechanisms. Int J Psychophysiol.

[41] Walker MP, van der Helm E (2009). Overnight therapy? The role of sleep in emotional brain processing. Psychol Bull.

[42] Pilcher JJ, Huffcutt AI (1996). Effects of sleep deprivation on performance: a meta-analysis. Sleep..

[43] Schwarz J, Axelsson J, Gerhardsson A, Tamm S, Fischer H, Kecklund G, Åkerstedt T (2019). Mood impairment is stronger in young than in older adults after sleep deprivation. J Sleep Res.

[44] Gerhardsson A, Fischer H, Lekander M, Kecklund G, Axelsson J, Åkerstedt T, Schwarz J (2019). Positivity effect and working memory performance remains intact in older adults after sleep deprivation. Front Psychol.

